# Reactive Oxygen Species and Autophagy Modulation in Non-Marine Drugs and Marine Drugs

**DOI:** 10.3390/md12115408

**Published:** 2014-11-13

**Authors:** Ammad Ahmad Farooqi, Sundas Fayyaz, Ming-Feng Hou, Kun-Tzu Li, Jen-Yang Tang, Hsueh-Wei Chang

**Affiliations:** 1 Laboratory for Translational Oncology and Personalized Medicine, Rashid Latif Medical College, Lahore 54000, Pakistan; E-Mails: ammadfarooqi@rlmclahore.com (A.A.F.); sundas.khan23@yahoo.com (S.F.); 2 Cancer Center, Kaohsiung Medical University Hospital; Kaohsiung Medical University, Kaohsiung 80708, Taiwan; E-Mail: mifeho@kmu.edu.tw; 3 Institute of Clinical Medicine, Kaohsiung Medical University, Kaohsiung 80708, Taiwan; 4 Kaohsiung Municipal Ta-Tung Hospital, Kaohsiung 80145, Taiwan; 5 Department of Biomedical Science and Environmental Biology, Kaohsiung Medical University, Kaohsiung 80708, Taiwan; E-Mail: sherry30126@yahoo.com.tw; 6 Department of Radiation Oncology, Kaohsiung Municipal Ta-Tung Hospital, Kaohsiung 80145, Taiwan; 7 Department of Radiation Oncology, Faculty of Medicine, College of Medicine, Kaohsiung Medical University, Kaohsiung 80708, Taiwan; 8 Department of Radiation Oncology, Kaohsiung Medical University Hospital, Kaohsiung 80708, Taiwan; 9 Institute of Medical Science and Technology, National Sun Yat-sen University, Kaohsiung 80424, Taiwan; 10 Research Center of Environmental Medicine, Kaohsiung Medical University, Kaohsiung 80708, Taiwan

**Keywords:** reactive oxygen species, autophagy, marine drugs, autophagy inhibitors, autophagy inducers

## Abstract

It is becoming more understandable that an existing challenge for translational research is the development of pharmaceuticals that appropriately target reactive oxygen species (ROS)-mediated molecular networks in cancer cells. In line with this approach, there is an overwhelmingly increasing list of many non-marine drugs and marine drugs reported to be involved in inhibiting and suppressing cancer progression through ROS-mediated cell death. In this review, we describe the strategy of oxidative stress-based therapy and connect the ROS modulating effect to the regulation of apoptosis and autophagy. Finally, we focus on exploring the function and mechanism of cancer therapy by the autophagy modulators including inhibitors and inducers from non-marine drugs and marine drugs.

## 1. Introduction

### 1.1. Strategy of Oxidative Stress-Based Therapy

Reactive oxygen species (ROS) are essential to regulate normal cellular processes. When excess ROS stimulation appear, it may trigger DNA repair responses in normal cells to remove the ROS-mediated DNA damage [1]. For highly active metabolism, cancer cells commonly have higher levels of ROS than normal cells [2], leading to carcinogenesis by oxidative DNA damage [3] and DNA repair impairment [1]. This nature of high ROS level in cancer cells also provides a chance for drug therapy to generate overloading ROS level and induce oxidative stress-induced cell death [2,4]. Therefore, the modulation of oxidative stress is a potential strategy to anticancer therapies [5].

## 2. Connection between ROS and Apoptosis in Marine Drugs

In this section, we described the protective function of ROS scavengers and apoptosis induction of ROS generating drugs of marine sources as follows:

### 2.1. Protective Function of ROS Scavengers of Marine Sources

Several marine natural products have proved to have an anti-oxidative effect [6]. For example, aqueous extracts of the edible *Gracilaria tenuistipitata* have demonstrated to protect against H_2_O_2_-induced plasmid and cellular DNA damage and reverted the H_2_O_2_-induced cytotoxicity of H1299 lung cancer cells [7]. Similarly, the brown alga *Sargassum horneri*-derived polysaccharides reportedly exert the protective effects against H_2_O_2_-induced injury in macrophage RAW264.7 cells. The results revealed that these biological effects were achieved by downregulating intracellular ROS, nitrogen oxide, and malonic dialdehyde (MDA) levels and by upregulating the level of antioxidant system (MnSOD and GSH-Px) in RAW264.7 cells [8]. Similarly, a lipid-soluble pigment of marine carotenoid astaxanthin can inhibit lipopolysaccaride-induced ROS generation and cytotoxicity via upregulation of superoxide dismutase (SOD) and catalase in mononuclear U937 cells [9].

### 2.2. Apoptosis Induction of ROS Generating Drugs of Marine Sources

In contrast, the accumulating evidence shows that several marine-derived extracts and compounds have the ROS inducible effects on different cancer cell lines. For example, 10-acetylirciformonin B (10AB), a marine sponge furanoterpenoid derived from irciformonin B [10], was reported to induce apoptosis via ROS generation in different cancer cell lines. Pretreatment of a ROS scavenger *N*-acetyl-l-cysteine to leukemia HL 60 cells drastically impaired 10AB-induced apoptosis, supporting that ROS generation was involved in irciformonin B-induced cytotoxicity of leukemia cells. Importantly, the protein expressions of Bcl-xL and Bcl-2, and caspase inhibitors (XIAP and surviving) were considerably repressed and the pro-apoptotic protein Bax was increased in 10AB treated leukemia HL 60 cells [11]. For another marine sponge derived compound Fascaplysin, it was apoptosis inducible in a chemoresistant NCI-H417 SCLC cells through ROS generation. Moreover, it was noted that fascaplysin worked synergistically with topoisomerase I-directed camptothecin and 10-hydroxy-camptothecin [12].

Dicitrinone B, a marine fungal metabolite, reportedly induced apoptosis via ROS generation in human malignant melanoma A375 cells. After pan-caspase inhibitor treatment to A375 cells, the dicitrinone B-induced ROS generation and apoptosis was abolished [13], suggesting that caspase pathway was involved in its ROS generation and apoptosis effects. A 48 kDa glycoprotein, isolated from a marine macroalga *Codium decorticatum*, was reported to induce ROS and apoptosis in breast cancer MDA-MB-231 cells through the intrinsic apoptosis pathway [14]. Surprisingly, it has been shown that lamellarin D, a marine alkaloid isolated from a marine mollusk *Lamellaria* sp. [15] and various ascidians [16], can induce ROS-mediated senescence in the absence of functional mitochondria in mouse leukemia P388 cells [17]. Marine triprenyl toluquinones and toluhydroquinones, originally purified from the Arminacean nudibranch *Leminda millecra*, have a similar ROS inducible effect to esophageal cancer WHCO1 cells [18]. Additionally, both methanolic extracts [19] and ethanolic extracts [20] of the editable red alga *Gracilaria tenuistipitata* showed the ROS generation and apoptosis induction in oral cancer cells. Ethyl acetate extracts from three marine algae (*Colpomenia sinuosa*, *Halimeda discoidae*, and *Galaxaura oblongata*) also displayed a ROS-mediated antiproliferative effect against human liver cancer and leukemia cells [21].

It had been reviewed that different natural products may induce different degrees of apoptosis and autophagy depending on their ROS modulating effect [22]. The marine drugs mentioned above have shown a ROS-mediated apoptotic effect, however, the possible roles of autophagy in these mechanisms warrant for further investigation.

## 3. Brief Introduction of Autophagy and Connection between ROS and Autophagy

In this section, we briefly introduce the autophagy and describe the relationship between ROS and autophagy as follows:

### 3.1. Brief Introduction of Autophagy

Autophagy is a “self-eating” behavior to ship cellular proteins and damaged organelles to lysosomes for recycling and it subsequently maintains the energy balance for cell survival during cell stress or starvation [23]. However, autophagy also reviewed to induce cell death in some cases [24]. There is a tremendously increasing amount of information regarding biology of autophagy. A substantial fraction of knowledge has been added into different steps of autophagy and it is now known that it is a highly regulated, multi-step molecular mechanism that initializes with induction, autophagosome nucleation, expansion and completion. Later steps of autophagy include lysosome fusion, degradation and recycling [25].

Structural studies have provided near complete resolution of protein network of mechanism of autophagy and mounting evidence suggested that initialization occurred through activation of AuTophaGy related 1 (Atg1) complex. It is multi-component machinery formed by assembly of Atg1, Atg13 and Atg17. Atg1 is a kinase that needs association of Atg13 and Atg17 for its activation. Vesicle nucleation is the subsequent process triggered by activation of the Vps34 and Beclin-1/Atg6. Autophagosome formation requires recruitment of proteins and lipids. Atg7 (E1-like) and Atg3 (E2-like) modulate vesicle elongation and completion by conjugation of phosphatidylethanolamine to microtubule-associated protein1 light chain 3 (LC3)/Atg8 which is initially processed by Atg4 [26]. Among them, the key step in autophagy is the proteolytic cleavage of LC3 to form LC3-I and subsequently modified to from LC3-II [27]. Moreover, Atg7 and Atg10 can join together to modulate the interaction between Atg12 and Atg5, and they are finally transferred to Atg16.

### 3.2. ROS May Lead to Autophagy

ROS are essential in maintaining normal cellular physiology, but ROS dysregulation may lead to tumor development and progression. Mitochondrial ROS generation play an important role for apoptosis and autophagy [25]. The autophagy may be induced to survival and cell death pathways in response to cellular oxidative stress [24]. Therefore, some of ROS-inducible drugs, such as 2-methoxyestradiol and arsenic trioxide, are used for cancer treatments [28]. Similarly, reduced scytonemin isolated from a terrestrial benthic cyanobacterium, *Nostoc commune,* induces ROS-based autophagy in human T-lymphoid Jurkat cells [29]. In the next sections, we will summarize many autophagy inhibitors and inducers derived from non-marine drugs and marine drugs to discuss the cancer therapy of those autophagy modulators.

## 4. Autophagy Inhibitors and Inducers from Non-Marine Drugs in Cancer Therapy 

Accumulating evidence showed that it was inter-compensatory between autophagy and apoptosis. For example, autophagy may have a cytotoxic role [30]. When the autophagy was induced, the cell death was promoted. For example, autophagic degradation of protein phosphatase Fap-1 was reported to enhance Fas-induced apoptosis. When cells displayed high autophagy, p62 recruited more Fap-1 for degradation and functional Fas ligands and receptors were highly maintained to activate more apoptotic signaling [31,32]. In this section, we described the function of the non-marine drugs derived autophagy inhibitors, autophagy inducers, clinical trial of autophagy inhibitor, clinical trial of autophagy inducers, and established anticancer drugs combined with autophagy inhibitors as follows:

### 4.1. Autophagy Inhibitors

Autophagy may have a cytoprotective role [30]. When the autophagy was inhibited, the cell death was promoted. For example, an autophagy inhibitor 3-methyladenine (3-MA) was reported to increase the apoptosis inducing potential of breast cancer MDA-MB 231 cells treated with a commercial mixture of tocotrienols and tocopherols (Tocomin^®^), which were isolated from palm oil/palm fruits [33,34]. It was revealed that mixture of tocotrienols and tocopherols can inhibit phosphoinositide 3-kinase (PI3K) and mammalian target of rapamycin (serine/threonine kinase) (mTOR) pathways, and induce the cytoprotective autophagic response in MDA-MB 231 cells, which could be overcome through inhibition of autophagy [34].

### 4.2. Autophagy Inducers

In accordance with the notion that Akt-mTOR signaling is a negative regulator of autophagy [35], gambogic acid, isolated from gamboge resin, can enhance the ROS accumulation and suppress phosphorylation of both Akt (S473) and mTOR (S2448) to induce autophagy in colorectal cancer HCT116 cells [36]. It is relevant to mention that extracellular signal-regulated kinases (ERK) pathway is also involved in initiation of autophagic response in hepatocellular carcinoma (HCC) cells as well as in mice xenografted with HCC cells [37]. A histone deacetylase inhibitor (HDACi) MGCD0103 has been shown to inhibit autophagy by functionalizing PI3K/AKT/mTOR pathway as well as caspases in B-cell chronic lymphocytic leukemia cells (CLL) [38]. Consistently, ATP-competitive mTOR kinase inhibitors (CC214-1 and CC214-2) were effective against rapamycin-resistant mTORC1 signaling to induce autophagy and prevent tumor cell death [39]. Cathepsin S, a lysosomal cysteine protease, was reported to overexpress in glioblastoma cells [40]. Inhibition of cathepsin S by its inhibitor Z-FL-COCHO (ZFL) can induce autophagy and mitochondrial-based apoptosis in glioblastoma cells. In autophagy-inhibitory glioblastoma cells by treating an autophagy inhibitor 3-MA or Beclin-1 shRNA, cathepsin S inhibition-induced apoptosis were drastically reduced. In cathepsin S-inhibitory glioblastoma cells, ROS-mediated PI3K/AKT/mTOR/p70S6K signaling pathway was inhibited and c-Jun N-terminal kinase (JNK) was activated [41].

### 4.3. Clinical Trial of Autophagy Inhibitors

Hydoxychloroquine (HCQ), a drug derived from quinolone, is antiproliferative to human dermal fibroblasts and induces autophagy in terms of upregulation of Beclin-1 [42,43]. Metastatic pancreatic cancer patients previously treated with HCQ at a dosage of 400 mg or 600 mg twice daily did not show considerable autophagy inhibition or therapeutic value [44]. Recently, the combined treatments of autophagy inhibitor HCQ with some drugs are being tested in preclinical and ongoing clinical cancer studies [45]. For example, HCQ is noted to effectively inhibit cancer growth in combination with epirubicin in xenografted mice [46]. However, the dosages of HCQ applied to inhibit autophagy are inconsistently functional in clinic studies [47].

Additionally, Lys05, a water-soluble salt of the lead compound Lys01 show that Lys05 targets to impair autophagy and inhibit tumor growth without toxicity under lower doses of Lys05 in mice studies [47]. These results suggest that Lys05 is warranted for further clinical trial in future.

### 4.4. Established Anticancer Drugs Combined with Autophagy Inhibitors

Emerging evidence has shed light on the fact that autophagy induced resistance against chemotherapeutic drugs in cancer cells, *i.e.*, a cytoprotective role of autophagy. In the following section we will discuss accumulating *in vitro* and *in vivo* evidence to understand how autophagy inhibition can be helpful in maximizing chemotherapeutic drug induced therapeutic effects in cancer cells. For example, treating with 3-MA or Beclin-1 siRNA to inhibit autophagy in colorectal cancer HCT116 and RKO cells, the low dose (20–50 nM) of a clinical drug for topoisomerase I inhibitor camptothecin-induced senescence was turned to caspase 3-dependent apoptosis [48]. For the combined treatment of clinical drugs sorafenib and vorinostat (the multikinase and HDAC inhibitors, respectively), its growth inhibitory efficacy can be enhanced in the autophagy inhibitor 3-MA treated hepatoma cells [49]. Inhibition of autophagy by beclin1 siRNA in ovarian cancer SKOV3/DDP cells has been noted to considerably increase cisplatin-induced apoptosis [50]. By pre-treatment of chloroquine for autophagy inhibition, DNA damaging agent 5-fluorouracil-induced cell death were remarkably increased in gallbladder carcinoma SGC-996 and GBC-SD cells [51]. Similarly, inhibition of autophagy by chloroquine can restore sensitivity of resistant lung cancer H3122CR-1 cells to crizonitib (PF02341066, the inhibitor of ALK fusion oncoprotein) [52].

Similar cytoprotective role of autophagy was also reported in literature. For example, overexpressing high-mobility group nucleosome-binding domain 5 (HMGN5) in osteosarcoma U2OS and MG63 cell lines can induce resistance against chemotherapeutic drugs such as doxorubicin, cisplatin, and methotrexate via inducing autophagy [53]. Inhibition of autophagy with clomipramine or metformin can enhance apoptosis and show the cytoprotective role of autophagy. Gene silencing with AMP-dependent protein kinase (AMPK) siRNA can substantially inhibit AMPK-induced downstream autophagy signaling and induce apoptosis in clinical trial drug enzalutamide (ENZA) treated prostate cancer cells. In mice orthotopically transplanted with ENZA-resistant cells, the combined treatment of ENZA and autophagy inhibitors (clomipramine and metformin) can reduce tumor growth compared to control groups [54]. The signal transducer and activator of transcription 3 (STAT3) was activated by oxidative stress. Downregulated STAT3 in pancreatic cancer cells also reported to inhibit cell growth through repressing autophagy induced by the treatment of Nexrutine(R) (Nx), a bark extract from *Phellodendron amurense* [55].

There is an exciting piece of evidence highlighting diametrically opposed role of autophagy as a pro-survival (cytoprotective), as well as a cell death-inducing (cytotoxic) role in cancer cells. For the example of cytotoxic role of autophagy, detailed investigation revealed that Akt activation and autophagy inhibition were responsible to the acquired resistance to sorafenib. A novel ATP-competitive pan-Akt inhibitor GDC0068 can reverse the acquired resistance to sorafenib, the first-line clinical drug for advanced HCC and autophagy was activated to be cytotoxic [56]. Similar cytotoxic role of autophagy was also reported that enforced expression of an imprinted tumor suppressor gene GTP-binding RAS-like 3 (DIRAS3 or ARHI) in DIRAS3-deficient ovarian cancer cells may induce autophagy and tumor dormancy [57]. In cells reconstituted with DIRAS3, growth factor-mediated intracellular signaling through PI3K and Ras/MAP kinase pathways were inhibited. Additionally, DIRAS3 can downregulate PI3K/AKT and Ras/ERK pathway and reduce phosphorylation of forkhead box O3 (FOXO3a) that facilitated transportation of FOXO3a to induce expression of autophagy-related genes (ATG4, MAP-LC3-I and Rab7) for maturation of autophagosomes and fusion with lysosomes [57]. Furthermore, DIRAS3 was reported to trigger assembly of autophagosome initiation complex to induce autophagy in dormant, nutrient-deprived ovarian cancer cells [58].

## 5. Autophagy Inhibitors and Inducers from Marine Drugs in Cancer Therapy 

As shown in Table 1, in this section we described the autophagy inhibitors and inducers of marine drugs of several species of the marine sponges, algae, bacteria/fungi/cyanobacteria, and other marine-derived compounds as follows:

**Table 1:** A list of bioactive ingredients that act as autophagy inhibitors and inducers.

Function	Marine Source	Source	Chemical	Target	References
Autophagy inhibitors	Marine Sponge	*Petrosaspongia nigra*	Petrosaspongiolide M	Beclin-1 ↓	[59,60] *
	Marine bacterium	*Streptomyces* spp.	Bafilomycins	LC3-II ↓	[61]
Autophagy inducers	Marine Sponge	*Haliclona* sp.	Manzamine A	LC3-II ↑ P62/SQTM1↑	[62] [63] *
		*Haliclona* sp.	Papuamine	LC3-II ↑	[64,65] *
		*Cliona celata*	Clionamines A–D	LC3 ↑	[66]
		*Geodia japonica*	Stellettin A	LC3-II ↑	[67,68] *
		*Rhabdastrella globostellata*	Rhabdastrellic acid-A	pAkt ↓	[69,70] *
	Alga	Green algae (*Enteromorpha intestinalis*; *Rhizoclonium riparium*)	Methanolic extracts	LC3-II ↑	[71]
		Red alga (*Laurencia dendroidea*)	Sesquiterpene elatol	endoplasmic reticulum extension ↑	[72] [73] *
		Brown algae	Fucoxanthin	LC3-II ↑ Beclin-1 ↑	[74] [75] *
	Marine bacterium/fungus/cyanobacterium	*Salinispora tropica; Salinispora arenicola*	Salinosporamide A	ATG5 ↑ ATG7 ↑	[76] [77] *
		*Chondrostereum* sp	Hirsutanol	LC3-II ↑ ROS↑	[78] [79] *
		*Penicillium commune*	SD118-xanthocillin X (1)	LC3-II ↑ mTOR, ERK ↓	[80]
		*Leptolyngbya* sp.	Coibamide	LC3-II ↑	[81]

* References state that autophagy-modulating drugs also have an apoptosis modulating effect.

### 5.1. Marine Sponge 

#### 5.1.1. Autophagy Inhibitors

##### 5.1.1.1. *Petrosaspongia* *Nigra*

Petrosaspongiolide M, a γ-hydroxybutenolide terpenoid isolated from a marine sponge *Petrosaspongia nigra* [82], can exert inhibitory effects on autophagy in human macrophage U937 cells in terms of downregulation of Beclin-1 level [59].

#### 5.1.2. Autophagy Inducers

##### 5.1.2.1. *Haliclona* sp.

Manzamine A, a kind of alkaloids for the uncoupler of vacuolar ATPases isolated from a marine sponge *Haliclona*, was reported to be a potential autophagy inducer. Mechanistically, manzamine A exerted its effects via increasing LC3-II and p62/SQSTM1 in pancreatic cancer cells [62]. Moreover, manzamine A can resensitize TRAIL-induced apoptosis in the pancreatic cancer AsPC-1 cells [63].

Papuamine, one of the isolated compounds from *Haliclona* sp. has been noted to decrease survival of breast cancer MCF-7 cells. Papuamine treated MCF-7 cells revealed an increase in expression of LC3 after 4 h treatment. Overall it suggested that papuamine induced early autophagy in MCF-7 cells that later activated JNK [64].

##### 5.1.2.2. *Cliona* *celata*

Aminosteroids clionamines A–D, isolated from South African sponge *Cliona celata*, was reported to induce autophagosome accumulation in terms of formation of cytoplasmic punctate Green Fluorescent Protein (GFP)-LC3 [66]. Clionamine B (2) was also reported to induce autophagy in human breast cancer MCF-7 cells [83].

##### 5.1.2.3. *Geodia* *japonica*

Stellettin, isolated from a marine sponge *Geodia japonica*, has been shown to induce autophagy in B16F10 murine melanoma cells. Increased LC3-II and its co-localization with tyrosinase indicated removal of deglycosylated and unfolded proteins [67].

##### 5.1.2.4. *Rhabdastrella* *globostellata*

Rhabdastrellic acid-A, an isomalabaricane Triterpenoid purified from a marine sponge *Rhabdastrella globostellata*, also notably induced autophagy in human lung cancer A549 cells. In Atg5 knockdown cells, rhabdastrellic acid-A mediated autophagy was impaired. pAkt was reduced in rhabdastrellic acid-A treated A549 cells and interestingly, transfecting constitutively active Akt in A549 cells can inhibit rhabdastrellic acid-A induced autophagy [69].

### 5.2. Alga

#### 5.2.1. Autophagy Inducers

##### 5.2.1.1. *Enteromorpha intestinalis* and *Rhizoclonium riparium*

Algal methanolic extracts from green alga *Enteromorpha intestinalis* and *Rhizoclonium riparium*, the saline/brackish water algae from Sundarbans, can induce autophagy in HeLa cells as evidenced by considerably enhanced expression of LC3-II [71].

#### 5.2.2. Laurencia dendroidea

Sesquiterpene elatol, the major bioactive compound of red seaweed *Laurencia dendroidea*, was reported to be an antiproliferative agent against *Leishmania amazonensis* with endoplasmic reticulum extension, which is an autophagy marker [72].

#### 5.2.3. Brown Algae

Fucoxanthin, a major carotenoid found in edible brown algae, was reported to be dose-responsively cytotoxic and G0/G1 arrest of HeLa cells without apoptosis change. Alternatively, autophagy-based cytotoxicity of fucoxanthin-treated HeLa cells was found involving the inhibition of Akt/mTOR signaling pathway [74].

### 5.3. Marine Bacterium/Fungus/Cyanobacterium

#### 5.3.1. Autophagy Inhibitors

##### 5.3.1.1. *Streptomyces* spp.

Eight bafilomycins (A1, B1, D, F, G, H, I, and J), purified from *Streptomyces* spp. of marine habitats, were proved to be potent inhibitors of autophagy in terms of automated microscopy screening assay-based punctate formation of EGFP-LC3 (autophagosome accumulation) and the Western blot-based EGFP-LC3 degradation assay [61]. Proteinase inhibitors, such as clasto-lactacystinblactone (LA) or epoxomicin (Epo) were recently reported to induce autophagy through inhibition of PI3K-Akt-mTOR pathway in human retinal pigment epithelial ARPE-19 cells [84]. Using the autophagy inhibitor bafilomycin A1, the protective effects of LA or Epo against menadione-induced oxidative injuries in ARPE-19 cells were reverted.

#### 5.3.2. Autophagy Inducers

##### 5.3.2.1. *Salinispora tropica* and *Salinispora arenicola*

Salinosporamide A, a potent proteasome inhibitor from marine bacteria *Salinispora tropica* and *Salinispora arenicola*, was reported to induce autophagy through a phospho-eukaryotic translation initiation factor 2α (eIF2α) pathway to reduce proteotoxic stresses in human prostate cancer cells [76].

##### 5.3.2.2. *Chondrostereum* sp.

Hirsutanol is a sesquiterpene isolated from marine fungus *Chondrostereum* sp. in the coral *Sarcophyton tortuosum* [85]*.* In hirsutanol-treated breast cancer MCF-7 cells, LC3-I to LC3-II conversion and ROS induction were markedly increased as evidenced by Western blot assay and flow cytometry [78].

##### 5.3.2.3. *Penicillium* *commune*

SD118-xanthocillin X, isolated from the marine fungus *Penicillium commune*, can induce autophagy in hepatocellular carcinoma HepG2 cells. There was a conversion of LC3-I to LC3-II, following lipidation as it incorporates into the nascent membrane of the autophagosome. Mechanistically it was noted that SD118-xanthocillin regulated different modulators of autophagy. It exerted its autophagy inducing effects via inhibition of phosphorylation of mTOR and ERK1/2. Additionally, Bcl-2 mediated inhibition of Beclin-1 to suppress autophagy was also attenuated via inhibition of Bcl-2 by SD118-xanthocillin [80].

##### 5.3.2.4. *Leptolyngbya* sp.

Coibamide A, a depsipeptide derived from marine cyanobacterium *Leptolyngbya* sp., showed a cytotoxicity in the dose-responsive and time-dependent manner in human glioblastoma cells and mouse embryonic fibroblasts (MEF) [81]. In coibamide A treated human glioblastoma U87-MG cells, LC3-II expression was notably increased. Coibamide A also induced the autophagosome accumulation in glioblastoma and MEF cells. Detailed mechanistic insights indicated that accumulation of autophagosomes was independent of mTOR-mediated signaling.

### 5.4. Other Marine-Derived Agents

Marine-derived agents, including eicosapentaenoic acid (EPA) and docosahexaenoic acid (DHA), are also potent inducers of autophagy as indicated by formation of autophagosomes in DHA- or EPA-treated lung adenocarcinoma A549 cells [86].

The Na^+^/K^+^-ATPases (NKA) inhibitor cardiac glycosides, a family of natural or synthetic steroid hormones isolated from marine or terrestrial natural products [87], can exert their potent anti-cancer properties via activation of Src in the upstream of MEK1/2 and ERK1/2 pathway in human non-small cell lung cancer A549 and H460 cells [88]. Src inhibition by its inhibitor PP2 or siRNA can remarkably repress cardiac glycosides-induced MEK1/2 and ERK1/2 phosphorylation and autophagic cell death. Moreover, ROS was also noted to be accumulated and contributed to cardiac glycosides-induced Src mediated autophagic response in lung cancer cells.

## 6. Conclusions

In this review, we summarized how ROS-mediated molecular networks may result in autophagy. The autophagic effects of both clinical drugs and natural products-derived extracts and pure compounds were discussed. In the example of many autophagy modulators (inducers and inhibitors) from non-marine drugs and marine drugs, ROS changes and signaling was demonstrated to be involved in autophagy. Many marine drugs with autophagy were also summarized from marine sponges, alga, and marine bacteria/fungi/ cyanobacteria. It suggests that marine drugs with ROS modulating effect have a potential to modulate the autophagy of cancer cells to improve cancer therapy.
